# Brain Connectivity and Network Analysis in Amyotrophic Lateral Sclerosis

**DOI:** 10.1155/2022/1838682

**Published:** 2022-02-07

**Authors:** Vijay Renga

**Affiliations:** Dartmouth Hitchcock Medical Center, Lebanon, NH 03756, USA

## Abstract

Amyotrophic lateral sclerosis (ALS) is a fatal neurodegenerative disease with no effective treatment or cure. ALS is characterized by the death of lower motor neurons (LMNs) in the spinal cord and upper motor neurons (UMNs) in the brain and their networks. Since the lower motor neurons are under the control of UMN and the networks, cortical degeneration may play a vital role in the pathophysiology of ALS. These changes that are not apparent on routine imaging with CT scans or MRI brain can be identified using modalities such as diffusion tensor imaging, functional MRI, arterial spin labelling (ASL), electroencephalogram (EEG), magnetoencephalogram (MEG), functional near-infrared spectroscopy (fNIRS), and positron emission tomography (PET) scan. They can help us generate a representation of brain networks and connectivity that can be visualized and parsed out to characterize and quantify the underlying pathophysiology in ALS. In addition, network analysis using graph measures provides a novel way of understanding the complex network changes occurring in the brain. These have the potential to become biomarker for the diagnosis and treatment of ALS. This article is a systematic review and overview of the various connectivity and network-based studies in ALS.

## 1. Introduction

Amyotrophic lateral sclerosis has an incidence of around 3/100,000. No definite etiology has been determined even though various genetic and environmental factors have been attributed [1]. ALS is mostly sporadic, but 5–10% of cases can be familial [2]. These are associated with SOD1, C9ORF, and other gene mutations that are still being identified. Postmortem studies have shown ALS to be a degenerative disorder of the anterior horn cells in the spinal cord and the cortical neurons, primarily in the motor cortex. The death of the neuronal cell body leads to degeneration of its axons and tracts leading to progressive disability and death [3–5]. In addition to axonal degeneration, there is hyperexcitability of surviving neurons and their networks. Loss of inhibitory neurons, increased glutamatergic activity, and functional reorganization secondary to increased compensation may underlie these changes [5–7]. In addition, interhemispheric inhibition is also impaired in ALS resulting in mirror movements [8].

The pattern of disease onset and rapidity of spread are highly variable. Progression ultimately results in respiratory failure and death, despite artificial means of life support. ALS is part of a spectrum of motor neuron disorders that includes frontotemporal dementia, primary lateral sclerosis, progressive muscular atrophy, and progressive bulbar palsy.

Due to the lack of biomarkers, it is often difficult to diagnose ALS in the early stages of the disease. Misdiagnosis is very common, and patients often go through various expensive and invasive tests before the condition declares itself. Even if diagnosed early, there are too few treatment options if any and that too only in prolonging life for a few months. The lack of biomarkers poses a big hurdle in monitoring response to novel treatments in ALS research.

The role of imaging findings as potential biomarkers gains importance in this scenario. Many structural and functional imaging studies have been performed over the years to assess their utility in ALS. There are no validated measures for clinical use yet. Newer studies using brain connectivity and network analysis look at the brain from a broader perspective as a connected network and have the potential to generate biomarkers for ALS. This article highlights the advances in imaging and electrophysiologic techniques to diagnose, assess severity, and predict the progression of ALS based on connectivity parameters and brain network analysis.

## 2. Methods

**Figure 1:**
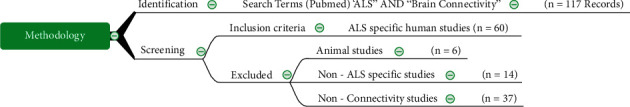
A total of 117 articles were obtained with the search terms. Only ALS-specific human imaging or electrophysiologic studies looking at connectivity were selected for this article. Animal studies, non-ALS studies, and nonconnectivity studies were excluded.

This article is a literature review of brain connectivity and network analysis studies in ALS (Table 1). Search Terms “ALS” and “Brain Connectivity” yielded 117 articles in Medline. Sixty ALS-specific human studies measuring connectivity and network analysis in ALS were selected for this article (see Figure 1).

The article also provides a primer on routine and specialized imaging in ALS, connectivity analysis, and generation and interpretation of networks using graph measures.

### 2.1. Clinical Imaging in ALS

Routine imaging studies done as part of clinical workup in ALS include CT scan and MRIs. The utility of the CT scan is limited to identifying focal or global atrophy of the brain and ruling out other pathologies or space-occupying lesions. MRI sequences used for clinical purposes include T1 and T2 weighted sequences, diffusion, and FLAIR images. Rarely, hyperintensities on FLAIR sequences are seen over the motor cortex in ALS patients that at times can be symmetric [9]. While useful as an additional diagnostic finding, these imaging abnormalities are rare and do not have any intrinsic value by themselves in the diagnosis or monitoring of ALS. The high-resolution anatomical MRI images can be further analyzed using voxel-based morphometry (VBM) or surface-based analysis to compute gray matter volumes (GMV). GMV loss is seen in motor and temporal cortices in ALS and frontotemporal dementia (FTD) patients [10]. A compensatory increase in volume in the cerebellar volume has also been reported [11].

### 2.2. Research Imaging in ALS

There are over 100 billion neurons in the brain that are interconnected through trillions of networks. Such levels of complexity are not measurable by histology or the current level of technology. Instead, imaging techniques such as fMRI, DTI, PET scan, and fNIRS and electrophysiologic techniques such as EEG and MEG can help us generate macroscale representations of these neuronal networks. With advances in imaging techniques, machine learning techniques, and large-scale studies such as the Human Connectome Project, it may become possible for us to define these connections in finer details.

Several brain connectivity studies have been done in ALS with some important and promising findings. However, results have varied and at times contradicted functional connectivity studies. Most studies in ALS have been multimodal with structural studies using high-resolution anatomical MRI and diffusion imaging for white matter tracts and functional connectivity studies using fMRI, EEG, PET, fNIRS, and MEG. The two most commonly used techniques are diffusion imaging and fMRI, which will be described further.

Connectivity studies can be based on structural or functional connectivity. Structural connectivity defines the anatomical connectivity between different regions of the brain. Typically, white matter tracts are defined using diffusion tensor imaging. Anatomical correlates can also be done using anatomical images and surface- or voxel-based techniques. Functional connectivity on the other hand establishes the electrophysiologic or metabolic functional correlates between different regions of the brain. EEG, PET scan, and time series correlates of fMRI data are used for this purpose.

### 2.3. Diffusion Imaging

Diffusion imaging captures the spatial diffusion of water molecules along the axonal white matter tracts in different spatial directions assuming a Gaussian distribution. Tensor-based analysis along white matter tracts can provide us fiber direction, axial, radial, and mean diffusivity as well as fractional anisotropy (FA). If the integrity of the myelin sheath is preserved, the fractional anisotropy and axial diffusivity are high, while the radial diffusivity is low. FA is reduced in conditions such as ALS where there is axonal degeneration.

**Figure 2:**
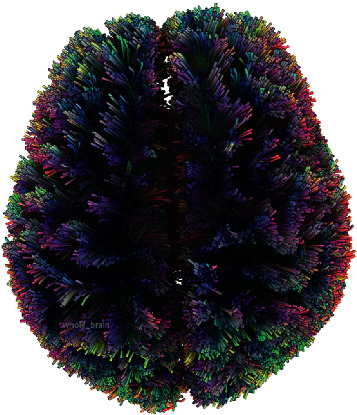
A whole-brain tractography generated based on Q-space diffusion imaging using DSI Studio. Color differences based on fiber direction.

Tractography, by fiber propagation, can generate a visual representation of white matter tracts that can then be further quantified. Deterministic tractography and probabilistic tractography are the two common techniques for generating the tracts. Deterministic tractography uses a set number of seed regions and parameters for propagation in a local fiber direction until a threshold is met for termination. Probabilistic tractography on the other hand takes into consideration propagation in any random direction. Both techniques have pros and cons. Newer techniques such as Q-space imaging may further improve the tracking of fiber bundles bypassing tissue edema and crossing fibers that are limitations of traditional DTI (Figure 2) [12, 13]. Preliminary studies using this technique have shown promising results in ALS that can quantify changes in white matter track volume during interval scans using a track difference paradigm [14, 15].

Once tracts are identified, network models can be generated for white matter connectivity. Tract disruptions in ALS are expected in the corticospinal tracts and corpus callosum, but a variety of other non-motor tracts and networks can be affected. Tract-based spatial statistics (TBSS) studies have shown reduced fiber density and reduced mean diffusivity along several white matter tracts in ALS [16–19].

Diffusion tensor imaging studies have consistently shown reduced fractional anisotropy of the corticospinal as well as non-motor tracts for both sporadic and familial ALS [18]. Large studies have also shown a correlation between FA values of corticospinal tracts (CST) with ALS Functional Rating Scale (ALSFRS scores) [20, 21]. Structural connectivity disruptions in the frontal lobe have also shown correlation with cognitive dysfunction in ALS patients [22].

### 2.4. Functional MRI

Functional MRI (fMRI) measures the changes in regional blood flow during resting or task activation. It uses the blood-oxygen-level-dependent (BOLD) contrast. As regional metabolism causes a shift from oxyhemoglobin to deoxyhemoglobin, there is a change in magnetization properties of blood that can be assessed using MRI. This can be a surrogate for local metabolism and neurovascular coupling [23]. It can also determine regions connected by similar metabolism during rest or activity, helping determine the interconnectedness of various regions using a time series analysis. Connectivity maps and network architecture can be then generated similar to structural connectivity.

## 3. Graph Theory and Brain Networks

Graph theory is a concept that is being increasingly applied to analyze complex brain networks [24]. This concept was first used by Leonard Euler in 1736 to solve the “Konigsberg Bridge” problem. Konigsberg city, which is now Kaliningrad in Russia, is set on both sides of the Pregel River. This created a mainland separated by two islands connected with seven brides. Euler was tasked to conceptualize a walkway throughout the landmasses that interconnected them in such a way that pedestrians needed to cross each bridge only once. Euler made a representation of each landmass as a node and the bridges connecting them as edges. Using early graph theory concepts, he proved that it was not possible to create such a walkway. Various network measures have since been defined to analyze and characterize network architecture. In the modern era, with the developments of transportation networks, the World Wide Web, and social networks, network-based studies became relevant again, and later, its adaptation to neuroscience as the brain is a highly complex network.

**Figure 3:**
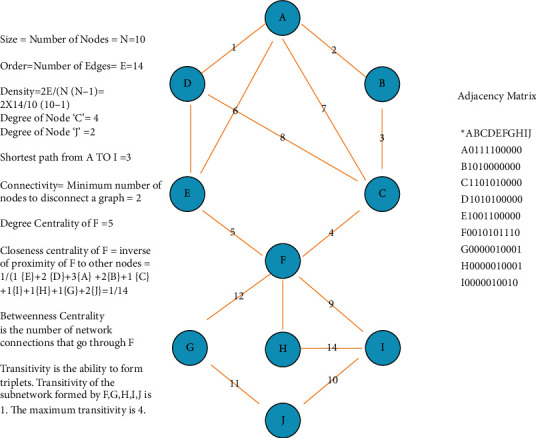
A simple unweighted, undirected graph and its adjacency matrix with some commonly used measures.

**Figure 4:**
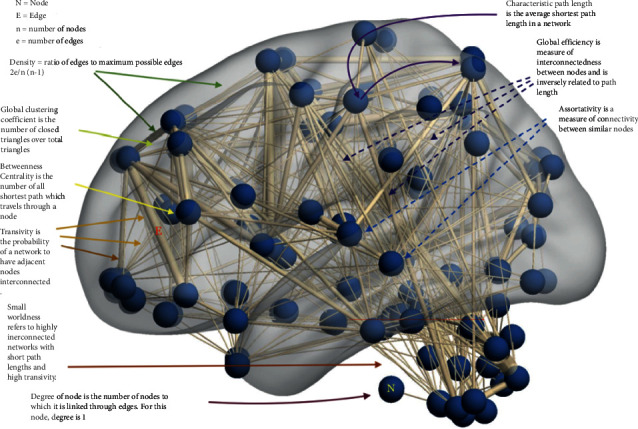
Various network measures used in graph theory analysis.

**Figure 5:**
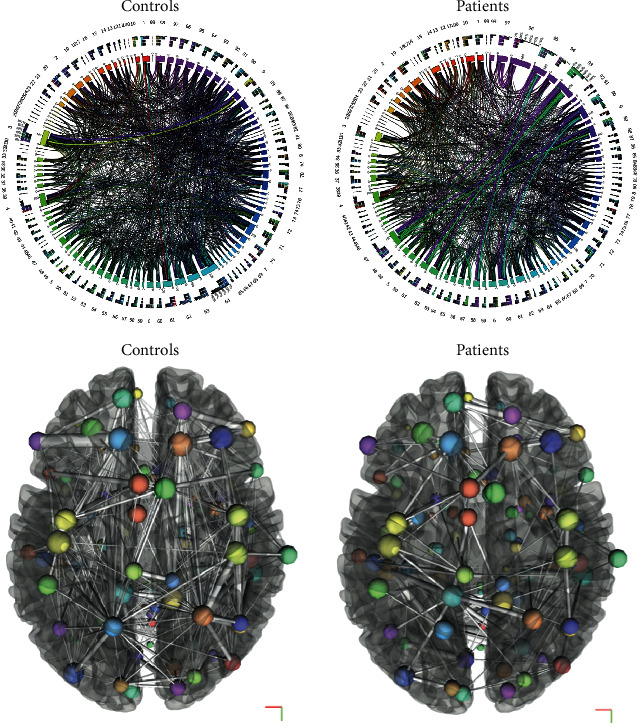
Averaged 2D connectograms and 3D structural networks of controls and patients (created with CIRCOS [78] and DSI studio [79])] showing reduced structural network connectivity in ALS. Both 2D and 3D models depict a reduced density of white matter connections in ALS patients (R) compared to controls (L). The patterns of connectivity changes can also be visualized.

Networks consist of nodes and edges. Edges can have directions (directed or undirected) and densities (weighted or unweighted). A simple binary unweighted, undirected graph architecture and various measures are depicted in the graph shown in Figure 2. Graphs can also be depicted using an adjacency matrix that depicts the connections between nodes and edges. It can also be represented in 3D models or 2D connectograms (Figures 3–5).

Brain networks can be created if we parcellate regions of brains into nodes and their connection as edges. Nodes could be anatomical regions, sites of electrode placement, or regions of interest and the “edges” derived from white matter tracts, electrical, hemodynamic, or metabolic connections or time series correlates connecting those nodes. High-resolution structural MRI images are generally used for nodes. Brain regions can be segmented into gray and white matters and then parcelled into distinct regions using anatomical atlases. Various atlases are available that can subdivide the cortical and subcortical regions anywhere from tens to as high as thousands of nodes.

Edges are generated from structural or functional connectivity parameters. Functional connectivity is generally assessed using fMRI time-series data from one region that correlates with other regions in terms of BOLD activity. This could be resting or dynamic. Structural connections are usually derived from diffusion imaging. The diffusion images are corrected for susceptibility and eddy current distortions. White matter edges can be generated using tractography techniques as described earlier.

Once the nodes and edges are defined, networks can be generated and a variety of graph measures are then applied to understand the brain architecture and its disruptions (Figure 4). Network-based statistical analysis can help individual or group of subjects [25]. Large-scale projects such as the Human Connectome Project can provide network databases and templates against which various disease processes could be compared and help understand the network derangement in a variety of diseases [26].

There are some complex graph measures as shown in (Figure 4). *Clustering coefficient* is the fraction of triangles around a node and is equivalent to the fraction of the node's neighbors that are neighbors of each other. It is a measure of nodes clustering together. *Transitivity* measures the probability that the adjacent nodes of a particular node are connected and is closely related to the clustering coefficient. It is calculated as a ratio of the observed number of closed triangles and the maximum possible number of closed triangles in the graph. High transitivity and low path lengths are characteristics of a small-world network. *Diameter* of a graph refers to the distance towards the maximally eccentric node and radius refers to the minimum eccentricity. The *efficiency* of a network refers to interconnectedness in a graph network. It can be global or regional. Global efficiency is inversely related to the path length in a network. *Assortativity* is a measure of similar nodes to be connected. *Rich club coefficients* refer to well-connected nodes that connect to each other.

## 4. Results

### 4.1. Connectivity Studies

Brain connectivity studies have utility in analyzing the anatomical and functional derangement in ALS [27]. They have the potential to become biomarkers for ALS [28, 29]. Longitudinal studies can define the evolution of structural and functional derangements and help us understand the underlying pathophysiology and progression of ALS [30].

Structural connectivity studies have unequivocally shown reduced fractional anisotropy along the motor and non-motor tracts [31–34]. The sites and degree of structural connectivity disruption have correlated with the rate of disease progression [35, 36]. Intra- and interhemispheric connectivity is also deranged in ALS [8, 20, 37]. This also holds true for genetic ALS with C9ORF mutations [18, 38, 39]. Local connectivity and network parameters vary between different types of motor neuron disease. Primary lateral sclerosis (PLS) and progressive muscular atrophy (PMA) can have differing patterns that can be helpful in identifying the subtype [40].

In contrast to structural connectivity studies, functional connectivity studies have shown differing results with some studies showing lowered and others showing increased functional connectivity in ALS, whether it be sporadic or genetic [41–43]. The pattern of reduced and increased functional connectivity in various regions of the brain also differs significantly. Patients with ALS were found to have reduced short-range functional connectivity density in the primary motor cortex and increased long-range connectivity in the premotor cortex [44]. Multimodal studies using anatomical (sMRI), diffusion (DTI), and resting fMRI scans have shown that the more structurally impaired networks overlapped with more functionally impaired connections [45]. Voxel mirrored homotopic interhemispheric connectivity of structural and functional networks involving the corpus callosum has shown reduced functional connectivity [46]. Decreased functional connectivity has been reported in the premotor cortex, corpus callosum, hippocampus, and cerebellar regions [11, 47–52]. In contrast to these findings, more studies have shown increased functional connectivity in ALS patients, even in regions with reduced structural connectivity [40, 53–58]. Dynamic connectivities of default motor networks and sensorimotor networks have increased connectivity in ALS [59]. Functional MRI (fMRI) studies using motor task activation have shown increased activation clusters in ALS patients compared to controls for the same task. Higher activation was seen in the prefrontal cortex in ALS patients compared to controls [60–63]. Such hyperconnectivity has been the predominant finding in studies using EEG, fNIRS, and MEG, even though some differences were noted here as well [53, 64–69]. Such differences in results can be explained by a heterogeneous progression of functional connectivity changes in ALS occurring at different stages of disease evolution when the patients were studied [44]. Functional hyperconnectivity or hyperexcitability may be the result of an intrinsic pathophysiologic process or a compensatory response to weakened musculature. Progressive increase in functional connectivity in frontoparietal and frontostriatal networks has been shown in longitudinal studies [70]. This increase in functional connectivity has also been shown to correlate with the severity of the disease process [67, 71–73]. Studies interrogating specific resting-state networks of the brain have identified increased connectivity in the default mode network and reduced connectivity in sensorimotor network at the same time and both these correlated to the severity of the disease process [74, 75]. While there are regions of increased and decreased regional functional connectivity in ALS, the global pattern across various studies leans towards increased functional connectivity, suggestive of the hyperexcitable cortex [59].

Despite several different studies, the functional connectivity changes in ALS remain complex and poorly defined. More longitudinal studies are needed to understand the evolution of functional connectivity disruption in ALS.

### 4.2. Network-Based Studies in ALS

Few studies have looked at network disruption in ALS using graph theory. These were mostly done using structural MRI (sMRI), fMRI, and DTI. Few EEG, MEG, and fNIRS studies have also been conducted.

Analysis of graph metrics using global measures in C9ORF mutation carriers showed lower global network density than healthy controls [43]. The mean clustering coefficient has been shown to be significantly higher in ALS patients compared to controls [68]. Resting-state functional networks in ALS versus healthy controls using a voxel-level approach showed significant differences in degree centrality in some regions in ALS [71]. The nodal degree in the left superior frontal region has also correlated with the ALSFRS-r scores.

Larger studies using high-resolution T1 weighted anatomical images and structural covariance networks showed significant increases in path length, clustering coefficient, small-world index, and local efficiency in ALS patients. At the same time, there was a significant decrease in global efficiency at several network densities in ALS patients compared to controls. Modularity was higher in ALS patients for several network densities. The modularity increase indicates fragmentation of brain architecture into more tightly clustered modules with poor intermodal communication in ALS patients. Changes were also noted in regional networks and network hubs. Higher functional connectivity strength hubs were seen in ALS patients compared to healthy controls [76]. Nodal betweenness was also increased in several regions in ALS suggesting an increase in information transfer across the available nodes suggestive of an increased “traffic.”

Significantly different networks connecting various regions of the brain have been found in ALS patients. Such findings are important to identify heterogeneously progressing vulnerable networks in ALS. Structural network analysis of these deranged networks showed reduced local and global efficiency in ALS patients compared to controls [40, 77]. Functional network studies in ALS have shown differing results like the functional connectivity studies. Verstraete et al. studied diffusion MRI-derived white matter structural connectome in patients with ALS. Subnetworks of significance were identified using network-based statistics [45, 56]. They detected impaired subnetworks within both motor cortex and distant regions, most of which were involved in motor control. In the impaired networks, they detected low network efficiency and density. A connectivity study done by the same group in ALS showed increased functional connectedness in ALS-affected structural networks [56]. A similar finding of impaired reduced structural connectivity within the prefrontal-motor-subcortical white matter network has been demonstrated using network-based statistics.

Electrophysiologic studies have also been useful in identifying the functional connectivity and network parameters in ALS patients. EEG data in ALS patients showed an increased spectral density of alpha bands. Clustering coefficients in alpha and gamma bands were increased in all regions of the scalp. Overall connectivity was increased in ALS patients with increased assortativity in the alpha band [68]. Resting-state MEG study in ALS also showed increased functional connectivity in the posterior cingulate cortex in ALS patients [53]. Functional network reorganization is perturbed in ALS, which has been shown to correlate with the disability [65, 69].

**Figure 6:**
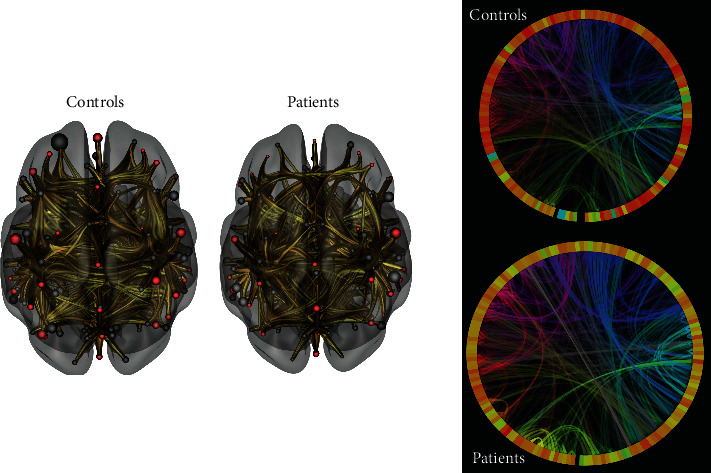
Averaged functional 2D connectogram and 3D networks of controls and patients (created using CONN Toolbox [80]). While 3D functional connection density appears similar, the pattern difference can be seen on the 2D connectograms. Analysis revealed increased interconnectedness (global efficiency) in ALS patients (not visualized in this picture).

We studied differences in global structural and functional connectivity patterns between eight ALS patients and eight age-matched healthy control data from online sources in a pilot connectivity in ALS (CoALS) study [54]. Global structural measures computed were density, clustering coefficient, transitivity, characteristic path length, small worldness, global efficiency, diameter, radius, assortativity, and rich club coefficients. For functional MRI global efficiency, local efficiency, betweenness centrality, average path length, clustering coefficient, and degree were compared between the groups. Structural network density was found to be significantly lower in ALS patients compared to control subjects (Figure 5). At the same time, the global functional efficiency was found to be significantly increased in ALS patients compared to control subjects (Figure 6). Other measures did not differ significantly. This finding is in alignment with our understanding of network architecture breakdown and hyperconnectivity in ALS. Bigger studies are underway to validate these findings and explore the network architecture further.

## 5. Conclusion

Brain connectivity and network analysis provide us with a novel and non-invasive approach to trace the progression of highly complex structural and functional derangements in ALS. Connectivity and network measures have shown correlation with disease progression and severity. While structural studies have unequivocally shown degrading connectivity and network architecture, functional studies have differed. These differences could be related to evolutionary changes occurring in a nonuniform pattern similar to the clinical presentation of ALS. More longitudinal studies are necessary to clarify this process.

The studies done so far have indicated a “traffic-jam” type pattern in the brain of ALS patients, where structural “road” networks are disrupted with an increase in functional connectedness or “traffic.” This functional interconnectedness and hyperexcitability seem to increase with the disease progression [67]. Whether this hyperexcitability is a response to structural disruption, or an intrinsic pathophysiologic process remains unclear [5, 81]. It can be hypothesized that a potential treatment of ALS would be the one that would either stop the structural network degradation or reduce the functional hyperconnectivity or both.

As we become more proficient in characterizing the underlying network changes with more standardized methodologies and automated analysis, we may be able to further characterize the intricate changes occurring in various stages of ALS [29, 82]. Vulnerable subnetworks, which are pathways of ALS progression, need to be isolated since those could become potential targets for treatment. Changing excitability of brain tissue using techniques like brain stimulation may be helpful in ALS [83].

Multimodal brain MRI findings using DTI and fMRI have already been proposed to have biomarker value for diagnosis and stratification in ALS [84]. Adding network analysis to these techniques may provide us with additional information to clarify and quantify responses to new and emerging treatments. With further refinements in connectivity analysis and a deeper understanding of the network topology, we may finally be able to define a network fingerprint for ALS that will have great implications in the diagnosis and treatment of ALS.

## Figures and Tables

**Table 1:** Connectivity and network studies done in ALS patients, sorted by year.

2021	Dynamic changes in functional network connectivity involving amyotrophic lateral sclerosis and its correlation with disease severity	Chen et al., 2021 [59]	32 ALS and 45 controls	sMRI and fMRI: Independent component analysis and dynamic functional network connectivity dFNC	ALS patients showed increased dFNC between DMN and SMN.
	Functional and structural impairment of transcallosal motor fibers in ALS: a study using transcranial magnetic stimulation, diffusion tensor imaging, and diffusion-weighted spectroscopy	Hübers et al., 2021 [8]	27 ALS and 21 controls	DTI and TMS: Fractional anisotropy (FA) interhemispheric inhibition (IHI)	ALS patients showed significantly decreased FA in the motor segment of the corpus callosum), and IHI was significantly reduced compared to controls.
	Segmental involvement of the corpus callosum in C9orf72-associated ALS: a Tract of interest-based DTI study	Müller et al., 2021 [18]	25 ALS and 25 controls	DTI: Tractwise fractional anisotropy statistics (TFAS)	Regional FA reduction for tracts of the Calloway areas II and III for ALS patients with C9orf72.
	Ultra-high field (7T) functional magnetic resonance imaging in amyotrophic lateral sclerosis: a Pilot study	Barry et al., 2021 [52]	12 ALS and 9 controls	sMRI and fMRI:	Reduced functional connectivity between the superior sensorimotor cortex and bilateral cerebellar lobule VI in ALS patients.
2020	Electrical and hemodynamic Neural Functions in People with ALS: an EEG-fNIRS resting-state study	Deligani et al., 2020 [64]	10 ALS and 9 controls	EEG and fNIRS: Connectivity study	Increased frontoparietal EEG connectivity in the alpha and beta bands and increased interhemispheric and right intrahemispheric fNIRS connectivity in the frontal and prefrontal regions were observed in ALS. Frontal, central, and temporal theta and alpha EEG power decreased in ALS, as did parietal and occipital alpha EEG power, while frontal and parietal hemodynamic spectral power increased in ALS.
	Frontal functional network disruption associated with amyotrophic lateral sclerosis: an fNIRS-based minimum spanning tree analysis	Borgheal et al., 2020 [65]	9 ALS and 10 controls	fNIRS: MST network analysis	Results showed significant between-group differences in several MST topological properties, including leaf fraction, maximum degree, diameter, eccentricity, and degree divergence.
	Multimodal longitudinal study of structural brain involvement in amyotrophic lateral sclerosis	van der Burgh et al., 2020 [38]	298 ALS and 156 controls	sMRI and DTI: cortical thickness, subcortical volumes, and white matter connectivity	Patients with a C9orf72 mutation showed widespread gray and white matter involvement at baseline and extensive loss of white matter integrity in the connectome over time. In C9orf72-negative patients, there was cortical thinning of motor and frontotemporal regions and loss of white matter integrity of connections linked to the motor cortex.
	Progression of brain functional connectivity and frontal cognitive dysfunction in ALS	Castelnovo et al., 2020 [70]	25 ALS longitudinal	FMRI: Resting-state functional connectivity changes	After six months, ALS patients showed an increased rsFC of the left anterior cingulate, left middle frontal gyrus (MFG), and left superior frontal gyrus within the frontostriatal network, left MFG, left supramarginal gyrus, and right angular gyrus within the left frontoparietal network.
	Regional callosal integrity and bilaterality of limb weakness in amyotrophic lateral sclerosis	Tu et al., 2020 [33]	29 ALS and 25 controls	DTI: Probabilistic tractography of the corpus callosum	In the combined patient group, the most prominent differences in diffusivity metrics were in the rostral body, posterior midbody, and isthmus of the corpus callosum. Loss of corpus callosum integrity was most prominent in the subgroup with unilateral limb weakness at the time of scanning.
	Resting-state functional connectivity is decreased globally across the C9orf72 mutation spectrum	Smallwood et al., 2020 [43]	25 ALS and 26 controls	FMRI: Graph theory analysis	Both global and connection-specific decreases in resting-state connectivity were observed, with no substantial reorganization of network hubs.
	Resting-state functional MRI brain signatures of fast disease progression in amyotrophic lateral sclerosis: a retrospective study	Trojsi et al., 2020 [51]	54 ALS and 22 controls	sMRI, DTI, and FMRI:	ALS patients showed reduced functional connectivity in both motor and extramotor networks.
	Structural and functional brain connectome in motor neuron diseases: a multicenter MRI study	Basia et a. 2020 [40]	179 ALS and 79 controls	sMRI, FMRI, and DTI: Connectivity and graph analysis	ALS and patients with PLS showed altered structural global network properties, as well as local topologic alterations and decreased structural connectivity in sensorimotor, basal ganglia, frontal, and parietal areas.
	Hippocampal connectivity in amyotrophic lateral sclerosis (ALS): more than Papez circuit impairment	Trojsi et al., 2020 [47]	32 ALS and 21 controls	fMRI and DTI: Seed-based connectivity (SBC), voxel-based morphometry (VBM), and tract-based spatial statistics (TBSS)	Decreased functional connectivity between bilateral hippocampus, bilateral parahippocampal gyri and cerebellum in ALS patients compared with HCs.
	Involvement of the dentate nucleus in the pathophysiology of amyotrophic lateral sclerosis: a multicenter and multimodal neuroimaging study	Bharti et al., 2020 [85]	71 ALS and 56 controls	sMRI, fMRI, and DTI: Seed- and RO-based connectivity and volumetric analysis	DN rsFC was reduced with cerebrum (supplementary motor area, precentral gyrus, frontal, posterior parietal, and temporal), lobule IV, and brain stem, and increased with the parieto-occipital region.
2019	Abnormal topological organization of structural covariance networks in amyotrophic lateral sclerosis	Zhang et al., 2019 [86]	60 ALS patients and 60 controls	sMRI: Structural covariance networks were studied in ALS patients and control subjects	Structural covariance networks of ALS patients showed an increased path length, clustering coefficient, small world index, and modularity, as well as decreased global efficiency and increased local segregation. Locally, ALS patients showed decreased nodal degree and betweenness in the gyrus rectus and/or Heschl's gyrus and increased betweenness in the supplementary motor area, triangular part of the inferior frontal gyrus, supramarginal gyrus, and posterior cingulate cortex. In addition, in ALS patients, there were more frontal and subcortical hubs than in normal controls.
	Characteristic increases in EEG connectivity correlate with changes of structural MRI in amyotrophic lateral sclerosis	Nasseroleslami et al., 2019 [87]	100 ALS and 34 controls	EEG + sMRi: EEG connectivity	Increased EEG coherence between parietal-frontal scalp regions (in *γ*-band) and between bilateral regions over motor areas (in *θ*-band).
	Patterned functional network disruption in amyotrophic lateral sclerosis	Dukic et al., 2019 [66]	74 ALS and 47 controls	EEG: Functional connectivity using beamformer source analysis	Decreased spectral power in the occipital and temporal (*δ*- to *β*-band), lateral/orbitofrontal (*δ*- to *θ*-band) and sensorimotor (*β*-band) regions of the brain in patients with ALS. Furthermore, we show increased comodulation of neural oscillations in the central and posterior (*δ*-, *θ*-, and *γ*l-band) and frontal (*δ*- and *γ*l-band) regions, as well as decreased synchrony in the temporal and frontal (*δ*- to *β*-band) and sensorimotor (*β*-band) regions.
	Precentral degeneration and cerebellar compensation in amyotrophic lateral sclerosis: a multimodal MRI analysis	Qiu et al., 2019 [11]	60 ALS and 60 controls	sMRI, fMRI, and DTI: Gray matter volume (GMV), white matter FA, and functional connectivity (FC)	Compared with healthy controls, patients with ALS showed decreased GMV in the left precentral gyrus and increased GMV in bilateral cerebellum, decreased FA in the left corticospinal tract and body of corpus callosum, and decreased FC in multiple brain regions, involving bilateral postcentral gyrus, precentral gyrus, and cerebellum anterior lobe, among others.
	Regional dynamics of the resting brain in amyotrophic lateral sclerosis using fractional amplitude of low-frequency fluctuations and regional homogeneity analyses	Bueno et al., 2019 [88]	20 ALS and 11 controls	fMRI: fractional amplitude of low-frequency fluctuations (fALFF) and regional homogeneity (ReHo)	Decreased fALFF and ReHO in ALS patients compared with HC in widespread cortical regions including motor and sensory regions.
	Structural connectivity alterations in amyotrophic lateral sclerosis: a graph theory-based imaging study	Fortainer et al., 2019 [77]	25 ALS and 26 controls	DTI: Structural connectivity	Patients with ALS exhibited global network alterations with decreased global efficiency (Eglob; *p* = 0.03) and a trend of the reduced whole-brain mean degree (*p* = 0.05) compared to controls.
2018	Abnormal functional connectivity density in amyotrophic lateral sclerosis	Li et al., 2018 [44]	38 ALS and 35 controls	fMRI: Functional connectivity density mapping (FCDM), an ultrafast data-driven method, that quantifies functional connections between a given voxel and all the other voxels in the entire brain	Patients with ALS were found to have decreased short-range FCD in the primary motor cortex and increased long-range FCD in the premotor cortex.
	Brain functional networks become more connected as amyotrophic lateral sclerosis progresses: a source level magnetoencephalographic study	Sorrentino et al., 2018 [67]	54 ALS and 25 controls	MEG: Betweenness centrality	The more advanced the disease, the more connected, scale-free, and disassortative the brain networks.
	Fast progressive lower motor neuron disease is an ALS variant: a two-centre tract of interest-based MRI data analysis	Müller et al., 2018 [35]	65 LMND, 92 controls, and 101 ALS	DTI: Fractional anisotropy maps and voxel-based comparison	Characteristic alteration patterns along the CST and also in frontal and prefrontal brain areas in LMND patients compared to controls and ALS. Fast progressing LMND showed substantial involvement, like in ALS, while slow progressors showed less severe alterations. FA of CST is affected in LMND just like ALS.
	Increased cerebral functional connectivity in ALS: a resting-state magnetoencephalography study	Proudfoot et al., 2018 [53]	24 ALS, 24 controls, 9 PLS, and 15 carriers	MEG: Power fluctuations in neuronal oscillation from distributed cortical parcels	Increased functional connectivity, particularly from the posterior cingulate cortex, was demonstrated in both patient groups compared to healthy controls.
	Monitoring value of multimodal magnetic resonance imaging in disease progression of amyotrophic lateral sclerosis: a prospective observational study	Shen et al., 2018 [89]	10 ALS longitudinal	sMRI, fMRI, DWI, and ASL: Multimodal imaging	Functional connectivity was increased in the motor areas (fALFF of the right precentral gyri and superior frontal gyri, and ReHo of right precentral gyri) and decreased in the extramotor areas.
	Regional thalamic MRI as a marker of widespread cortical pathology and progressive frontotemporal involvement in amyotrophic lateral sclerosis	Tu et al., 2018 [34]	20 ALS and 31 controls	DTI: Diffusion tractography to parcellate the thalamus	Widespread diffusivity alterations in motor and extramotor associated thalamic parcellations.
	The two-year progression of structural and functional cerebral MRI in amyotrophic lateral sclerosis	Menke et al., 2018 [90]	16 ALS longitudinal	sMRI, DTI, and FMRI: VBM and independent component analysis (ICA)	Widespread and progressive reductions in gray matter were observed in the precentral gyri and posterior cingulate cortex, as well as progressive local atrophy of the thalamus, caudate, and pallidum bilaterally, and right putamen, hippocampus, and amygdala. The most prominent DTI tract-based changes were in the superior longitudinal fasciculus and corpus callosum. FC decreases were noted between the sensorimotor resting-state network and the frontal pole, between a network comprising both thalami and an area in the visual cortex.
	Unraveling ALS due to SOD1 mutation through the combination of brain and cervical cord MRI	Agosta et al., 2018 [39]	31 ALS and 33 controls.	sMRI, fMRI, DTI: Cortical thickness analysis, diffusion tensor MRI of the corticospinal tracts (CST) and corpus callosum, and resting-state functional connectivity	Fractional anisotropy showed that sporadic ALS patients had significant CST damage relative to both healthy controls and SOD1-related ALS although the latter showed alterations that were intermediate between controls and sporadic ALS. Functional hyperconnectivity of the motor cortex in the sensorimotor network was observed in patients with sporadic ALS relative to controls.
2017	Aberrant interhemispheric homotopic functional and structural connectivity in amyotrophic lateral sclerosis	Zhang et al., 2017 [46]	38 ALS and 35 controls.	fMRI and DTRI: Voxel mirrored homotopic connectivity (VMHC) and probabilistic fiber tracking that quantifies functional connectivity between each voxel in one hemisphere and its mirrored counterpart in the opposite hemisphere	Extensive reductions of VMHC associated with ALS in brain regions of the precentral and postcentral gyrus, the paracentral lobule, the superior temporal gyrus, the middle cingulate gyrus, the putamen, and the superior parietal lobules. With DTI, the analysis has also revealed reductions of interhemispheric structural connectivity through the CC subregions II, III, and V in patients with ALS. Additionally, interhemispheric functional connectivity of the bilateral precentral gyri positively correlated with fractional anisotropy values of the CC subregion III, which structurally connects the bilateral motor cortices.
	Brain functional connectome abnormalities in amyotrophic lateral sclerosis are associated with disability and cortical hyperexcitability	Geevasinga et al., 2017 [72]	20 ALS and 20 controls	sMRI, fMRI and TMS: Using threshold tracking transcranial magnetic stimulation (TMS) and functional connectivity	Increased functional connectivity in 12 network edges connecting 14 nodes. Connectivity changes in frontal regions are inversely correlated with functional disability. The mean clustering coefficient was significantly increased in patients with ALS.
	Frequency-specific abnormalities of Intrinsic functional connectivity strength among patients with amyotrophic lateral sclerosis: a resting-state fMRI study	Li et al., 2017 [91]	21 ALS and 21 controls	fMRI: Functional connectivity strength (FCS)	ALS patients showed a significantly decreased FCS in the left prefrontal cortex (PFC) and the bilateral superior frontal gyrus. FCS changes in ALS were widespread and frequency-dependent.
	Resting-state fMRI correlates of theory of mind impairment in amyotrophic lateral sclerosis	Trojsi et al., 2017 [50]	21 ALS and 15 controls	fMRI: Resting-state connectivity	Decreased connectivity in frontotemporal areas within the main cognitive resting-state networks, including the default mode (DMN), the right and left frontoparietal (R-, L-FPN), and the salience (SLN) networks, in the entire ALS group.
	White matter structural network abnormalities underlie executive dysfunction in amyotrophic lateral sclerosis	Dimond et al., 2017 [22]	18 ALS and 22 controls	DTI: Tract-based statistics	ALS cognitive impaired patients displayed altered local connectivity and structural integrity in these same frontal regions that correlated with executive dysfunction.
2016	A large-scale multicenter cerebral diffusion tensor imaging study in amyotrophic lateral sclerosis	Müller et al., 2016 [92]	253 ALS and 189 controls	DTI study: Fractional anisotropy (FA) maps were used	Significant difference in CST, frontal lobe, brainstem, and hippocampus in ALS patients. These changes correlated with the postmortem neuropathologic changes.
	Corticoefferent pathways in pure lower motor neuron disease: a diffusion tensor imaging study	Rosenbohm et al., 2016 [31]	37 LMND and 53 controls	DTI: FA, AD, RD, MC, diffusion tractography, and whole-brain spatial statistics	The analysis demonstrated widespread and characteristic patterns of alterations in patients with LMND, predominantly along the corticospinal tract (CST), with multiple clusters of regional FA reductions.
	Disrupted effective connectivity of the sensorimotor network in amyotrophic lateral sclerosis	Fang et al., 2016 [27]	20 ALS patients and 21 controls	fMRI: Dynamic causal modeling analysis	In ALS patients, significant regional activity alterations in the left primary motor cortex (M1), the left primary somatosensory cortex (S1) and the right supplementary motor cortex (SMA) are found. Among these regions, spectral DCM revealed a missing closed-loop circuit between the left M1 and the right SMA and lost projection from the right SMA to the left S1 in ALS.
	EEG functional network topology is associated with disability in patients with amyotrophic lateral sclerosis	Fraschini et al., 2016 [69]	21 ALS and 16 controls	EEG: Functional connectivity using the phase lag index (PLI) and network topology using the minimum spanning tree (MST)	No significant group differences were observed for the global mean PLI in any frequency band. A significant MST dissimilarity between ALS patients and healthy controls was found in the beta band.
	Functional connectivity changes resemble patterns of pTDP-43 pathology in amyotrophic lateral sclerosis	Schulthess et al., 2016 [55]	135 ALS and 56 controls	fMRI and DTI	Functional connectivity patterns associated with the motor, brainstem, ventral attention, and default mode/hippocampal intrinsic connectivity network revealed increased connectivity maps in ALS patients.
	Increased functional connectivity common to symptomatic amyotrophic lateral sclerosis and those at genetic risk	Menke et al., 2016 [57]	12 ALS and 12 controls	sMRI, fMRI, and DWI: Cortical thickness analysis, voxel-based morphometry, volumetric and shape analyses of subcortical structures, tract-based spatial statistics of metrics derived from the diffusion tensor, and resting-state functional connectivity (FC) analyses were performed	Gray matter cortical thickness and shape analysis revealed significant atrophy in patients with ALS (but not psALS) compared with controls in the right primary motor cortex and right caudate. Comparison of diffusion tensor metrics showed widespread fractional anisotropy and radial diffusivity differences in patients with ALS compared to controls and the psALS group, encompassing parts of the corpus callosum, corticospinal tracts, and superior longitudinal fasciculus. While FC in the resting-state sensorimotor network was similar in psALS and controls, FC between the cerebellum and a network comprising the precuneus, cingulated, and middle frontal lobe was significantly higher in psALS and affected ALS compared to controls.
	Investigating default mode and sensorimotor network connectivity in amyotrophic lateral sclerosis	Chenji et al., 2016 [74]	21 ALS and 40 controls	fMRI: Compared to default mode networks and sensorimotor networks	Significant group differences in resting-state networks between patients and controls were absent, as was the dependence on the degree of UMN burden. However, DMN connectivity was increased in patients with greater disability and faster progression rate, and SMN connectivity was reduced in those with greater motor impairment.
	Occipital cortical gyrification reductions associate with decreased functional connectivity in amyotrophic lateral sclerosis	Zhang et al., 2016 [49]	25 ALS and 25 controls.	sMRI and fMRI: Surface-based local gyrification index (LGI) and seed-based functional connectivity	ALS patients had significantly reduced LGI in the right occipital cortex, and that abnormality in this region was associated with decreased functional connectivity in the bilateral precuneus.
	Widespread temporo-occipital lobe dysfunction in amyotrophic lateral sclerosis	Loawe et al., 2016 [41]	64 ALS and 38 controls	FMRI: whole-brain functional connectivity graph analysis	Clusters of reduced functional connectivity were observed in the sensorimotor cortex.
2015	Altered cortical hubs in functional brain networks in amyotrophic lateral sclerosis	Ma et al., 2015 [76]	20 ALS and 20 HC	fMRI: Functional connectivity strength (FCS)	High FCS hubs in the prefrontal cortex in ALS versus healthy controls.
	Cerebro-cerebellar connectivity is increased in primary lateral sclerosis	Meoded et al., 2015 [73]	16 PLS patients and 14 controls	fMRI and DTI: Structural and functional connectivity	PLS patients had 12 regions of increased functional connectivity.
	Functional connectivity changes in resting-state EEG as potential biomarker for amyotrophic lateral sclerosis	Iyer et al., 2015 [68]	18 ALS and 17 controls	EEG: Graph theory measures	Cross spectral density in the alpha band was higher in patients. In ALS patients, increased degree values of the network nodes were noted in the central and frontal regions in the theta band across seven of the different connectivity maps, among patients, the clustering coefficient in alpha and gamma bands was increased in all regions of the scalp and connectivity were significantly increased (*p* = 0.02). The nodal network showed increased assortativity in the alpha band in the patients group. The clustering coefficient in partial directed connectivity (PDC) showed significantly higher values for patients in alpha, beta, gamma, theta, and delta frequencies.
	Metabolic spatial connectivity in amyotrophic lateral sclerosis as revealed by independent component analysis	Pagani et al., 2015 [28]	259 ALS and 40 controls.	PET: Spatial ICA	Eight components were selected as pathophysiologically meaningful discriminated patients from controls with 99.0% accuracy.
	Reduced structural connectivity within a prefrontal-motor-subcortical network in amyotrophic lateral sclerosis	Buchanan et al., 2015 [32]	30 ALS and 30 controls	sMRI and DTI: Network-based statistics	Impaired motor-frontal-subcortical subnetwork (10 nodes and 12 bidirectional connections), consistent with upper motor neuron pathology. Reduced FA in three of the impaired network connections.
2014	Additional resources and the default mode network: evidence of increased connectivity and decreased white matter integrity in amyotrophic lateral sclerosis	Heimrath et al., 2014 [75]	9 ALS patients and 11 controls	fMRI and DTI: Study focused on verbal fluency and attention and the relationship with functional connectivity in the DMN and white matter integrity	Patients showed increased functional connectivity in parahippocampal and parietal areas of the non-task-associated DMN compared to controls. The more pronounced the cognitive deficits, the stronger the increase in functional connectivity in those areas. White matter integrity was reduced in frontal areas in the patients. In conclusion, increased connectivity in the DMN in parahippocampal and parietal areas might represent the recruitment of accessory brain regions to compensate for dysfunctional frontal networks.
	Correlation between structural and functional connectivity impairment in amyotrophic lateral sclerosis	Schmidt et al., 2014 [45]	64 ALS and 27 controls	sMRI, DWI, and fMRI	(i) The most structurally affected connections considerably overlap with the most functionally impaired connections; (ii) direct connections of the motor cortex are both structurally and functionally more affected than connections at a greater topological distance from the motor cortex; and (iii) there is a strong positive correlation between changes in SC and FC averaged per brain region (r50.44, *P* < 0.0001).
	Structural brain network imaging shows expanding disconnection of the motor system in amyotrophic lateral sclerosis	Verstraete et al., 2014 [21]	24 ALS and 22 controls	DTI: Whole-brain tractography	Demonstrated an expanding subnetwork of affected brain connections over time with a central role for the primary motor regions.
2013	Altered motor network functional connectivity in amyotrophic lateral sclerosis: a resting-state functional magnetic resonance imaging study	Zhou et al., 2013 [93]	12 ALS patients and 12 controls	fMRI: Functional connectivity (FC) of motor cortex	Both decreased and increased within-motor network FC in ALS patients. Increased FC between the bilateral superior parietal lobule and the right anterior inferior cerebellum was found to be correlated with disease severity, with higher FC related to more severe disease.
	Divergent brain network connectivity in amyotrophic lateral sclerosis	Agosta et al., 2013 [42]	20 ALS and 15 controls	fMRI: Independent component analysis	ALS patients showed a decreased connectivity of the right orbitofrontal cortex and enhanced connectivity of the left precuneus in the default mode network; a decreased connectivity of the left inferior frontal cortex and increased connectivity of the right angular gyrus in the right frontoparietal network; and increased connectivity of the parietal cortex in the left frontoparietal network.
	Dysfunctions within limbic-motor networks in amyotrophic lateral sclerosis	Passmonti et al., 2013 [60]	11 ALS and 12 HC	fMRI: fMRI task analysis	ALS patients had greater activation in the PFC areas and altered left amygdala PFC connectivity.
	Multiple kernel learning captures a systems-level functional connectivity biomarker signature in amyotrophic lateral sclerosis	Feket et al., 2013 [48]	40 ALS and 30 controls	fMRI: Intra- and intermotor functional network analysis	ALS patients had reduced connectivity of both the cortical and subcortical motor areas with non-motor areas, reduced subcortical-cortical motor connectivity, and increased connectivity observed within subcortical motor networks.
	The utility of independent component analysis and machine learning in the identification of the amyotrophic lateral sclerosis diseased brain	Welsh et al., 2013 [29]	32 ALS and 31 controls	fMRI: ICA analysis and support vector machine (SVM) machine learning analysis	Over 71% accuracy for disease state classification is obtained.
2012	Direct evidence of intra- and interhemispheric corticomotor network degeneration in amyotrophic lateral sclerosis: an automated MRI structural connectivity study	Rose et al., 2012 [20]	15 ALS and 15 HC	sMRI and DWI: Whole-brain diffusion tractography	Significant reduction in mean FA within a number of intra- and interhemispheric motor pathways in ALS patients is noted.
2011	Impaired structural motor connectome in amyotrophic lateral sclerosis	Verstraete et al., 2011 [37]	35 ALS and 19 controls	sMRI and DTI: Fiber tracking and network analysis	ALS patient had impaired subnetwork of regions with reduced white matter connectivity around primary motor regions (bilateral precentral gyrus and right paracentral lobule), including secondary motor regions (bilateral caudal middle frontal gyrus and pallidum) as well as high-order hub regions (right posterior cingulate and precuneus). In addition, a significant reduction in overall efficiency and clustering is noted.
	Sensorimotor functional connectivity changes in amyotrophic lateral sclerosis	Agosta et al., 2011 [58]	26 ALS and 15 controls	fMRI: Resting functional connectivity analysis	ALS patients versus controls showed a significantly increased functional connectivity between the left SMC and the right cingulate cortex, parahippocampal gyrus, and cerebellum-crus II.
2010	Motor network degeneration in amyotrophic lateral sclerosis: a structural and functional connectivity study	Verstaete et al., 2010 [56]	12 ALS and 12 controls	sMRI, DTI, and fMRI: Whole-brain cortical thickness and diffusion tensor imaging (DTI) of crucial motor tracts and functional connectivity analysis of the motor network based on resting-state fMRI	Functional organization of the motor network was unchanged in patients with ALS compared to healthy controls; however, the level of functional connectedness was significantly correlated with disease progression rate. Patients with increased connectedness had progressive disease courses.
2006	Probabilistic diffusion tractography: a potential tool to assess the rate of disease progression in amyotrophic lateral sclerosis	Ciccarelli et al., 2006 [36]	13 ALS and 19 controls	DTI: Probabilistic tractography, FA analysis of connectivity along CST, and disease progression rate	Patients with rapid progression had a significantly lower mean FA.

Notes: sMRI: structural MRI; fMRI: functional MRI; DTI: diffusion tensor imaging; EEG: electroencephalogram; fNIRS: functional near-infrared spectroscopy; PET: positron emission tomography.

## References

[1] Andrew A. S., Bradley W. G., Peipert D. (2021). Risk factors for amyotrophic lateral sclerosis: a regional United States case‐control study. *Muscle & Nerve*.

[2] Chiò A., Calvo A., Moglia C., Mazzini L., Mora G., PARALS study group (2011). Phenotypic heterogeneity of amyotrophic lateral sclerosis: a population based study. *Journal of Neurology Neurosurgery and Psychiatry*.

[3] Kiernan J. A., Hudson A. J. (1991). Changes in sizes of cortical and lower motor neurons in amyotrophic lateral sclerosis. *Brain*.

[4] Nihei K., McKee A. C., Kowall N. W. (1993). Patterns of neuronal degeneration in the motor cortex of amyotrophic lateral sclerosis patients. *Acta Neuropathologica*.

[5] Kohara N. (1999). Abnormal hyperexcitability in ALS. *Rinsho Shinkeigaku*.

[6] Menon P., Kiernan M. C., Vucic S. (2015). Cortical hyperexcitability precedes lower motor neuron dysfunction in ALS. *Clinical Neurophysiology*.

[7] Van den Bos M. A. J., Higashihara M., Geevasinga N., Menon P., Kiernan M. C., Vucic S. (2018). Imbalance of cortical facilitatory and inhibitory circuits underlies hyperexcitability in ALS. *Neurology*.

[8] Hübers A., Böckler B., Abaei A. (2021). Functional and structural impairment of transcallosal motor fibres in ALS: a study using transcranial magnetic stimulation, diffusion tensor imaging, and diffusion weighted spectroscopy. *Brain Imaging and Behavior*.

[9] Zhang L., Ulu A. M., Zimmerman R. D., Lin M. T., Rubin M., Beal M. F. (2003). The diagnostic utility of FLAIR imaging in clinically verified amyotrophic lateral sclerosis. *Journal of Magnetic Resonance Imaging*.

[10] Bede P., Omer T., Finegan E. (2018). Connectivity-based characterisation of subcortical grey matter pathology in frontotemporal dementia and ALS: a multimodal neuroimaging study. *Brain Imaging and Behavior*.

[11] Qiu T., Zhang Y., Tang X. (2019). Precentral degeneration and cerebellar compensation in amyotrophic lateral sclerosis: a multimodal MRI analysis. *Human Brain Mapping*.

[12] Yeh F.-C., Wedeen V. J., Tseng W.-Y. I. (2010). Generalized ${ q}$-sampling imaging. *IEEE Transactions on Medical Imaging*.

[13] Yeh F.-C., Verstynen T. D., Wang Y., Fernández-Miranda J. C., Tseng W.-Y. I. (2013). Deterministic diffusion fiber tracking improved by quantitative anisotropy. *PLoS One*.

[14] Abhinav K., Yeh F. C., El-Dokla A. (2014). Use of diffusion spectrum imaging in preliminary longitudinal evaluation of amyotrophic lateral sclerosis: development of an imaging biomarker. *Frontiers in Human Neuroscience*.

[15] Yeh F.-C., Zaydan I. M., Suski V. R. (2019). Differential tractography as a track-based biomarker for neuronal injury. *NeuroImage*.

[16] Smith S. M., Jenkinson M., Johansen-Berg H. (2006). Tract-based spatial statistics: voxelwise analysis of multi-subject diffusion data. *NeuroImage*.

[17] Alruwaili A. R., Pannek K., Coulthard A., Henderson R., Kurniawan N. D., McCombe P. (2018). A combined tract-based spatial statistics and voxel-based morphometry study of the first MRI scan after diagnosis of amyotrophic lateral sclerosis with subgroup analysis. *Journal of Neuroradiology*.

[18] Müller H.-P., Lulé D., Roselli F., Behler A., Ludolph A. C., Kassubek J. (2021). Segmental involvement of the corpus callosum in C9orf72-associated ALS: a tract of interest-based DTI study. *Therapeutic Advances in Chronic Disease*.

[19] Ciccarelli O., Behrens T. E., Johansen-Berg H. (2009). Investigation of white matter pathology in ALS and PLS using tract-based spatial statistics. *Human Brain Mapping*.

[20] Rose S., Pannek K., Bell C. (2012). Direct evidence of intra- and interhemispheric corticomotor network degeneration in amyotrophic lateral sclerosis: an automated MRI structural connectivity study. *NeuroImage*.

[21] Verstraete E., Veldink J. H., van den Berg L. H., van den Heuvel M. P. (2014). Structural brain network imaging shows expanding disconnection of the motor system in amyotrophic lateral sclerosis. *Human Brain Mapping*.

[22] Dimond D., Ishaque A., Chenji S. (2017). White matter structural network abnormalities underlie executive dysfunction in amyotrophic lateral sclerosis. *Human Brain Mapping*.

[23] Nair D. G. (2005). About being BOLD. *Brain Research Reviews*.

[24] Rubinov M., Sporns O. (2010). Complex network measures of brain connectivity: uses and interpretations. *NeuroImage*.

[25] Zalesky A., Fornito A., Bullmore E. T. (2010). Network-based statistic: identifying differences in brain networks. *NeuroImage*.

[26] Van Essen D. C., Smith S. M., Barch D. M., Behrens T. E. J., Yacoub E., Ugurbil K. (2013). The Wu-Minn human connectome project: an overview. *NeuroImage*.

[27] Fang X., Zhang Y., Wang Y. (2016). Disrupted effective connectivity of the sensorimotor network in amyotrophic lateral sclerosis. *Journal of Neurology*.

[28] Pagani M., Öberg J., De Carli F. (2016). Metabolic spatial connectivity in amyotrophic lateral sclerosis as revealed by independent component analysis. *Human Brain Mapping*.

[29] Welsh R. C., Jelsone-Swain L. M., Foerster B. R. (2013). The utility of independent component analysis and machine learning in the identification of the amyotrophic lateral sclerosis diseased brain. *Frontiers in Human Neuroscience*.

[30] Menke R. A. L., Proudfoot M., Talbot K., Turner M. R. (2018). The two-year progression of structural and functional cerebral MRI in amyotrophic lateral sclerosis. *NeuroImage: Clinical*.

[31] Rosenbohm A., Müller H.-P., Hübers A., Ludolph A. C., Kassubek J. (2016). Corticoefferent pathways in pure lower motor neuron disease: a diffusion tensor imaging study. *Journal of Neurology*.

[32] Buchanan C. R., Pettit L. D., Storkey A. J., Abrahams S., Bastin M. E. (2015). Reduced structural connectivity within a prefrontal-motor-subcortical network in amyotrophic lateral sclerosis. *Journal of Magnetic Resonance Imaging*.

[33] Tu S., Wang C., Menke R. A. L. (2020). Regional callosal integrity and bilaterality of limb weakness in amyotrophic lateral sclerosis. *Amyotrophic Lateral Sclerosis and Frontotemporal Degeneration*.

[34] Tu S., Menke R. A. L., Talbot K., Kiernan M. C., Turner M. R. (2018). Regional thalamic MRI as a marker of widespread cortical pathology and progressive frontotemporal involvement in amyotrophic lateral sclerosis. *Journal of Neurology, Neurosurgery & Psychiatry*.

[35] Müller H. P., Agosta F., Riva N. (2018). Fast progressive lower motor neuron disease is an ALS variant: a two-centre tract of interest-based MRI data analysis. *NeuroImage. Clinical*.

[36] Ciccarelli O., Behrens T. E., Altmann D. R. (2006). Probabilistic diffusion tractography: a potential tool to assess the rate of disease progression in amyotrophic lateral sclerosis. *Brain*.

[37] Verstraete E., Veldink J. H., Mandl R. C. W., van den Berg L. H., van den Heuvel M. P. (2011). Impaired structural motor connectome in amyotrophic lateral sclerosis. *PLoS One*.

[38] van der Burgh H. K., Westeneng H.-J., Walhout R. (2020). Multimodal longitudinal study of structural brain involvement in amyotrophic lateral sclerosis. *Neurology*.

[39] Agosta F., Spinelli E. G., Marjanovic I. V. (2018). Unraveling ALS due to SOD1 mutation through the combination of brain and cervical cord MRI. *Neurology*.

[40] Basaia S., Agosta F., Cividini C. (2020). Structural and functional brain connectome in motor neuron diseases. *Neurology*.

[41] Loewe K., Machts J., Kaufmann J. (2017). Widespread temporo-occipital lobe dysfunction in amyotrophic lateral sclerosis. *Scientific Reports*.

[42] Agosta F., Canu E., Valsasina P. (2013). Divergent brain network connectivity in amyotrophic lateral sclerosis. *Neurobiology of Aging*.

[43] Smallwood Shoukry R. F., Clark M. G., Floeter M. K. (2020). Resting state functional connectivity is decreased globally across the C9orf72 mutation spectrum. *Frontiers in Neurology*.

[44] Li W., Zhang J., Zhou C. (2018). Abnormal functional connectivity density in amyotrophic lateral sclerosis. *Frontiers in Aging Neuroscience*.

[45] Schmidt R., Verstraete E., Reus M. A., Veldink J. H., den Berg L. H., den Heuvel M. P. (2014). Correlation between structural and functional connectivity impairment in amyotrophic lateral sclerosis. *Human Brain Mapping*.

[46] Zhang J., Ji B., Hu J. (2017). Aberrant interhemispheric homotopic functional and structural connectivity in amyotrophic lateral sclerosis. *Journal of Neurology Neurosurgery and Psychiatry*.

[47] Trojsi F., Di Nardo F., Caiazzo G. (2021). Hippocampal connectivity in amyotrophic lateral sclerosis (ALS): more than papez circuit impairment. *Brain Imaging and Behavior*.

[48] Fekete T., Zach N., Mujica-Parodi L. R., Turner M. R. (2013). Multiple Kernel learning captures a systems-level functional connectivity biomarker signature in amyotrophic lateral sclerosis. *PLoS One*.

[49] Zhang Y., Fang T., Wang Y. (2017). Occipital cortical gyrification reductions associate with decreased functional connectivity in amyotrophic lateral sclerosis. *Brain Imaging and Behavior*.

[50] Trojsi F., Di Nardo F., Santangelo G. (2017). Resting state fMRI correlates of theory of mind impairment in amyotrophic lateral sclerosis. *Cortex*.

[51] Trojsi F., Di Nardo F., Siciliano M. (2021). Resting state functional MRI brain signatures of fast disease progression in amyotrophic lateral sclerosis: a retrospective study. *Amyotrophic Lateral Sclerosis and Frontotemporal Degeneration*.

[52] Barry R. L., Babu S., Anteraper S. A. (2021). Ultra-high field (7T) functional magnetic resonance imaging in amyotrophic lateral sclerosis: a pilot study. *NeuroImage: Clinical*.

[53] Proudfoot M., Colclough G. L., Quinn A. (2018). Increased cerebral functional connectivity in ALS. *Neurology*.

[54] Renga V. (2020). Connectivity in ALS (CoALS)—a pilot study (359). *Neurology. Wolters Kluwer Health, Inc. on behalf of the American Academy of Neurology*.

[55] Schulthess I., Gorges M., Müller H.-P. (2016). Functional connectivity changes resemble patterns of pTDP-43 pathology in amyotrophic lateral sclerosis. *Scientific Reports*.

[56] Verstraete E., van den Heuvel M. P., Veldink J. H. (2010). Motor network degeneration in amyotrophic lateral sclerosis: a structural and functional connectivity study. *PLoS One*.

[57] Menke R. A. L., Proudfoot M., Wuu J. (2016). Increased functional connectivity common to symptomatic amyotrophic lateral sclerosis and those at genetic risk. *Journal of Neurology, Neurosurgery & Psychiatry*.

[58] Agosta F., Valsasina P., Absinta M. (2011). Sensorimotor functional connectivity changes in amyotrophic lateral sclerosis. *Cerebral Cortex*.

[59] Chen H.-J., Zou Z.-Y., Zhang X.-H., Shi J.-Y., Huang N.-X., Lin Y.-J. (2021). Dynamic changes in functional network connectivity involving amyotrophic lateral sclerosis and its correlation with disease severity. *Journal of Magnetic Resonance Imaging*.

[60] Passamonti L., Fera F., Tessitore A. (2013). Dysfunctions within limbic-motor networks in amyotrophic lateral sclerosis. *Neurobiology of Aging*.

[61] Kollewe K., Münte T. F., Samii A., Dengler R., Petri S., Mohammadi B. (2011). Patterns of cortical activity differ in ALS patients with limb and/or bulbar involvement depending on motor tasks. *Journal of Neurology*.

[62] Mohammadi B., Kollewe K., Samii A., Krampfl K., Dengler R., Münte T. F. (2009). Decreased brain activation to tongue movements in amyotrophic lateral sclerosis with bulbar involvement but not Kennedy syndrome. *Journal of Neurology*.

[63] Mohammadi B., Kollewe K., Samii A., Dengler R., Münte T. F. (2011). Functional neuroimaging at different disease stages reveals distinct phases of neuroplastic changes in amyotrophic lateral sclerosis. *Human Brain Mapping*.

[64] Deligani R. J., Hosni S. I., Borgheai S. B. (2020). Electrical and hemodynamic neural Functions in people with ALS: an EEG-fNIRS resting-state study. *IEEE Transactions on Neural Systems and Rehabilitation Engineering*.

[65] Borgheai S. B., McLinden J., Mankodiya K., Shahriari Y. (2020). Frontal functional network disruption associated with amyotrophic lateral sclerosis: an fNIRS-based minimum spanning tree analysis. *Frontiers in Neuroscience*.

[66] Dukic S., McMackin R., Buxo T. (2019). Patterned functional network disruption in amyotrophic lateral sclerosis. *Human Brain Mapping*.

[67] Sorrentino P., Rucco R., Jacini F. (2018). Brain functional networks become more connected as amyotrophic lateral sclerosis progresses: a source level magnetoencephalographic study. *NeuroImage: Clinical*.

[68] Iyer P. M., Egan C., Pinto-Grau M. (2015). Functional connectivity changes in resting-state EEG as potential biomarker for amyotrophic lateral sclerosis. *PLoS One*.

[69] Fraschini M., Demuru M., Hillebrand A. (2016). EEG functional network topology is associated with disability in patients with amyotrophic lateral sclerosis. *Scientific Reports*.

[70] Castelnovo V., Canu E., Calderaro D. (2020). Progression of brain functional connectivity and frontal cognitive dysfunction in ALS. *NeuroImage: Clinical*.

[71] Zhou C., Hu X., Hu J. (2016). Altered brain network in amyotrophic lateral sclerosis: a resting graph theory-based network study at voxel-wise level. *Frontiers in Neuroscience*.

[72] Geevasinga N., Korgaonkar M. S., Menon P. (2017). Brain functional connectome abnormalities in amyotrophic lateral sclerosis are associated with disability and cortical hyperexcitability. *European Journal of Neurology*.

[73] Meoded A., Morrissette A. E., Katipally R., Schanz O., Gotts S. J., Floeter M. K. (2015). Cerebro-cerebellar connectivity is increased in primary lateral sclerosis. *NeuroImage: Clinical*.

[74] Chenji S., Jha S., Lee D. (2016). Investigating default mode and sensorimotor network connectivity in amyotrophic lateral sclerosis. *PLoS One*.

[75] Heimrath J., Gorges M., Kassubek J. (2014). Additional resources and the default mode network: evidence of increased connectivity and decreased white matter integrity in amyotrophic lateral sclerosis. *Amyotrophic Lateral Sclerosis and Frontotemporal Degeneration*.

[76] Ma X., Zhang J., Zhang Y. (2015). Altered cortical hubs in functional brain networks in amyotrophic lateral sclerosis. *Neurological Sciences*.

[77] Fortanier E., Grapperon A.-M., Le Troter A. (2019). Structural connectivity alterations in amyotrophic lateral sclerosis: a graph theory based imaging study. *Frontiers in Neuroscience*.

[78] Krzywinski M. I., Schein J. E., Birol I. Circos: an information aesthetic for comparative genomics. *Genome Research*.

[79] http://dsi-studio.labsolver.org/course/apparent-diffusion-coefficient-and-the-tensor-model.

[80] Whitfield-Gabrieli S., Nieto-Castanon A. (2012). Conn: a functional connectivity toolbox for correlated and anticorrelated brain networks. *Brain Connectivity*.

[81] Bakulin I. S., Chervyakov A. V., Suponeva N. A., Zakharova M. N., Piradov M. A. (2016). Motor cortex hyperexcitability, neuroplasticity, and degeneration in amyotrophic lateral sclerosis. *Update on Amyotrophic Lateral Sclerosis*.

[82] Tomasi D., Volkow N. D. (2010). Functional connectivity density mapping. *Proceedings of the National Academy of Sciences*.

[83] Benussi A., Alberici A., Cotelli M. S. (2019). Cortico-spinal tDCS in ALS: a randomized, double-blind, sham-controlled trial. *Brain Stimulation*.

[84] Mazón M., Vázquez Costa J. F., Ten-Esteve A., Martí-Bonmatí L. (2018). Imaging biomarkers for the diagnosis and prognosis of neurodegenerative diseases. The example of amyotrophic lateral sclerosis. *Frontiers in Neuroscience*.

[85] Bharti K., Khan M., Beaulieu C. (2020). Involvement of the dentate nucleus in the pathophysiology of amyotrophic lateral sclerosis: a multi-center and multi-modal neuroimaging study. *NeuroImage: Clinical*.

[86] Zhang Y., Qiu T., Yuan X. (2019). Abnormal topological organization of structural covariance networks in amyotrophic lateral sclerosis. *NeuroImage: Clinical*.

[87] Nasseroleslami B., Dukic S., Broderick M. (2019). Characteristic increases in EEG connectivity correlate with changes of structural MRI in amyotrophic lateral sclerosis. *Cerebral Cortex*.

[88] Bueno A. P. A., Pinaya W. H. L., Rebello K., de Souza L. C., Hornberger M., Sato J. R. (2019). Regional dynamics of the resting brain in amyotrophic lateral sclerosis using fractional amplitude of low-frequency fluctuations and regional homogeneity analyses. *Brain Connectivity*.

[89] Shen D.-C., Xu Y.-Y., Hou B. (2018). Monitoring value of multimodal magnetic resonance imaging in disease progression of amyotrophic lateral sclerosis. *Chinese Medical Journal*.

[90] Menke R. A. L., Proudfoot M., Talbot K., Turner M. R. (2017). The two-year progression of structural and functional cerebral MRI in amyotrophic lateral sclerosis. *NeuroImage Clin*.

[91] Li F., Zhou F., Huang M., Gong H., Xu R. (2017). Frequency-specific abnormalities of intrinsic functional connectivity strength among patients with amyotrophic lateral sclerosis: a resting-state fMRI study. *Frontiers in Aging Neuroscience*.

[92] Müller H. P., Turner M. R., Grosskreutz J. (2016). A large-scale multicentre cerebral diffusion tensor imaging study in amyotrophic lateral sclerosis. *Journal of Neurology Neurosurgery and Psychiatry*.

[93] Zhou F., Gong H., Li F. (2013). Altered motor network functional connectivity in amyotrophic lateral sclerosis. *NeuroReport*.

